# An economic evaluation of Alexander Technique lessons or acupuncture sessions for patients with chronic neck pain: A randomized trial (ATLAS)

**DOI:** 10.1371/journal.pone.0178918

**Published:** 2017-12-06

**Authors:** Holly Essex, Steve Parrott, Karl Atkin, Kathleen Ballard, Martin Bland, Janet Eldred, Catherine Hewitt, Ann Hopton, Ada Keding, Harriet Lansdown, Stewart Richmond, Helen Tilbrook, David Torgerson, Ian Watt, Aniela Wenham, Julia Woodman, Hugh MacPherson

**Affiliations:** 1 Department of Health Sciences, University of York, York, United Kingdom; 2 Society of Teachers of the Alexander Technique, London, United Kingdom; 3 British Acupuncture Council, London, United Kingdom; 4 Sydera Research Associates, Market Weighton, York, United Kingdom; 5 Hull York Medical School, University of York, York, United Kingdom; University of Toronto, CANADA

## Abstract

**Objectives:**

To assess the cost-effectiveness of acupuncture and usual care, and Alexander Technique lessons and usual care, compared with usual GP care alone for chronic neck pain patients.

**Methods:**

An economic evaluation was undertaken alongside the ATLAS trial, taking both NHS and wider societal viewpoints. Participants were offered up to twelve acupuncture sessions or twenty Alexander lessons (equivalent overall contact time). Costs were in pounds sterling. Effectiveness was measured using the generic EQ-5D to calculate quality adjusted life years (QALYs), as well as using a specific neck pain measure–the Northwick Park Neck Pain Questionnaire (NPQ).

**Results:**

In the base case analysis, incremental QALY gains were 0.032 and 0.025 in the acupuncture and Alexander groups, respectively, in comparison to usual GP care, indicating moderate health benefits for both interventions. Incremental costs were £451 for acupuncture and £667 for Alexander, mainly driven by intervention costs. Acupuncture was likely to be cost-effective (ICER = £18,767/QALY bootstrapped 95% CI £4,426 to £74,562) and was robust to most sensitivity analyses. Alexander lessons were not cost-effective at the lower NICE threshold of £20,000/QALY (£25,101/QALY bootstrapped 95% CI -£150,208 to £248,697) but may be at £30,000/QALY, however, there was considerable statistical uncertainty in all tested scenarios.

**Conclusions:**

In comparison with usual care, acupuncture is likely to be cost-effective for chronic neck pain, whereas, largely due to higher intervention costs, Alexander lessons are unlikely to be cost-effective. However, there were high levels of missing data and further research is needed to assess the long-term cost-effectiveness of these interventions.

## Introduction

Neck pain is a leading cause of disability worldwide [1], representing a substantial economic burden due to healthcare resources consumed and wider costs to society through productivity losses [2]. As chronic neck pain is difficult to manage [3], it is not unusual for people with chronic pain to pursue complementary healthcare options [4, 5].

Acupuncture involves localised insertion of needles, often accompanied by diagnostic explanations and advice regarding lifestyle [6–8]. A trial has found acupuncture to be effective for chronic neck pain when compared to usual care over a three month period [9]. A recent individual patient data meta-analysis of high quality trials found acupuncture to be effective for chronic back and neck pain, osteoarthritis and headache/migraine [10] Acupuncture has also been found to be cost-effective treatment for some chronic pain conditions [11]. The UK’s National Institute for Health and Care Excellence (NICE) endorsed acupuncture as a referral option for low back pain and headache/migraine in 2009 and 2012, respectively [12, 13]. There are currently no such recommendations with regard to neck pain. Recent evidence on acupuncture for people with chronic neck pain has found health benefits at 12 months, however the extent that this intervention is cost-effective is unknown [14].

The Alexander Technique is a taught self-care method that helps people enhance their control of reaction and improve their way of going about everyday activities. It is usually taught through one-to-one practical lessons involving integrated didactic and hands-on implicit guidance, that enable people to reduce habits associated with musculoskeletal pain [15]. Applying the Technique in daily life is associated with improved postural tone, balance, coordination and motor control [16–19], whilst health benefits include long-term improvements in chronic low-back pain [15, 20] and associated cost-effectiveness [21]. Alexander Technique lessons have been found in a recent trial to be beneficial for people with chronic neck pain at 12 months, however the cost-effectiveness of this intervention is also unknown [14].

This study reports an economic evaluation conducted alongside a one-year randomized controlled trial (RCT) of Alexander Technique lessons or acupuncture sessions (ATLAS), compared with usual care, for people with chronic, non-specific neck pain. Our objectives were to determine the value for money and effectiveness of these strategies and estimate the incremental cost effectiveness of Alexander lessons or acupuncture over and above usual care.

## Methods

### The ATLAS study

Full details of the trial are published elsewhere [14, 22]. Briefly, the ATLAS (Alexander Technique Lessons or Acupuncture Sessions) study was a pragmatic, multi-centre, three-armed RCT comparing acupuncture or Alexander lessons with usual GP care alone, for people with chronic, non-specific neck pain. Participants were recruited from general practitioner (GP) surgeries in York, Sheffield, Leeds and Manchester, UK. Screening of surgery databases identified potential participants who had experienced neck pain for 3 months or more and scored ≥28% (10/36 points for car drivers, 9/32 for non-drivers) on the Northwick Park neck pain (and associated disability) Questionnaire (NPQ) [23]. Patients were excluded if they were currently receiving acupuncture for neck pain or had attended Alexander lessons in the previous two years, were pursuing litigation, or had any of a number of underlying health conditions (serious underlying pathology, prior cervical spine surgery, history of psychosis, rheumatoid arthritis, ankylosing spondylitis, osteoporosis, hemophilia, cancer, HIV or hepatitis, ongoing or recent alcohol or drug dependency). Patients were also excluded if they were pregnant at study onset (due to higher risk of loss to follow-up), involved in another trial that may conflict with the current study, or if they were unable to speak English. Ethical approval was obtained from Leeds West Research Ethics Committee and all participants provided written informed consent. The trial was prospectively registered as number ISRCTN15186354 on the International Standardised Randomised Controlled Trial Number registry.

### Randomisation

Participants were randomly allocated to one of the three groups using a secure randomisation computer system. Participants randomised to the acupuncture arm were offered up to twelve 50-minute sessions (total time 600 minutes) of acupuncture from a British Acupuncture Council (BAcC) member. Participants in the Alexander arm were offered up to twenty 30-minute one-to-one lessons (total time 600 minutes) with an Alexander teacher registered with the Society of Teachers of the Alexander Technique (STAT). Participants in the both the acupuncture and Alexander arms also continued to receive GP care as usual. Both interventions were typically offered weekly at the outset (twice weekly for Alexander if desired), and fortnightly later, with delivery time usually over a 5 month period, as intended.

### Economic viewpoint

Two perspectives were considered. As recommended by NICE [24], the primary analysis was conducted from a UK National Health Service (NHS) perspective. In addition, a broader societal perspective captured costs to patients from private healthcare and productivity losses due to neck pain.

### Resource use measurement

Self-report postal questionnaires at baseline, six and 12 months collected data on the resources used by each participant during the trial period, both specifically relating to neck pain and in general. Participants were asked to recall NHS healthcare received in the previous six-month period including: appointments with a GP, practice nurse, physiotherapist, hospital visits including outpatient appointments, day case and other admissions, accident and emergency visits and the use of prescription medication. Data were also collected on private healthcare sought, including acupuncture sessions, Alexander lessons and other private care for neck pain, and the amount paid. Finally, data were collected on days off work due to neck pain, as well as annual income data, to estimate productivity losses. Details of the interventions were recorded by Alexander teachers and acupuncturists using log books.

### Unit costs

Resources used in the study were valued using national average unit costs, at 2012/13 prices. As NHS cost sources do not include complementary healthcare practitioners, the costs of Alexander Technique lessons and the acupuncture sessions were valued at a per session cost based on the rate paid to the practitioners taking part in the study, reflective of national rates [25], a method adopted in previous studies [21, 26]. Table 1 provides a summary of the key unit cost estimates used. To estimate cost of the interventions, quantities of services used were multiplied by the relevant unit cost in order to estimate overall cost profiles for trial participants. To cost the interventions, session rates were multiplied by the actual number of sessions attended by each participant in the trial. As trial follow-up occurred 12 months post-randomisation, no discounting was required as costs and consequences were captured over a one year time horizon.

**Table 1:** Unit costs used in the trial (2012/13 prices).

Resource type	Item of resource	Unit	Unit cost (£)	Source
Intervention	Alexander Technique^a^	Lesson	33.00	ATLAS trial
	Acupuncture^b^	Session	35.00	ATLAS trial
NHS	General practitioner	11.7 minute surgery appointment	34.00	Curtis [27]
	Practice nurse	15.5 minute surgery appointment	11.37	Curtis [27]
	Physiotherapist^c^	Appointment	36.17	Curtis [27], Department of Health [28]
	Hospital outpatient	Visit	108.00	Department of Health [28]
	Accident and Emergency	Visit	115.00	Department of Health [28]
	Hospital day case^d^	Visit	693.00	Department of Health [28]
	Other hospital admission	Admission	1,877.86	Department of Health [28]
	Prescription	Prescription item	8.37	Health & Social Care Information Centre [29]

^a^20 lessons offered (600 minutes total)

^b^12 sessions offered (600 minutes total)

^c^Based on an average of physiotherapist appointments in primary and secondary care settings

^d^Planned admission

### Outcome measures

Quality adjusted life years (QALYs) were estimated based on participants’ responses to the EuroQol EQ-5D-3L questionnaire [30] collected at baseline, six and 12 months. The EQ-5D is a generic utility measure of health-related quality of life which assesses current health states across five dimensions: mobility, self-care, usual activities, pain/discomfort, and anxiety/depression with three levels: none, some or extreme problems. Questionnaire responses to the first section of the EQ-5D were converted to a single summary index score using UK preference valuations of different combinations of the EQ-5D dimensions [31], and these scores were, in turn, converted to QALYs using the area under the curve (AUC) method [32].

Utility measures can be relatively insensitive to smaller changes in health status [33]. The Northwick Park Neck Pain Questionnaire (NPQ) [23] was therefore used as a secondary outcome measure. The NPQ is a nine-item measure of neck pain and associated disability and was the primary clinical effectiveness measure for the trial. The NPQ scale was converted to a percentage score (0–100) where 0 represents no neck pain or disability and 100 represents maximum neck pain or disability. The outcome for the economic analysis was expressed as a change in scores between baseline and 12 months.

### Analysis

The primary analysis was an incremental cost effectiveness (or cost utility) analysis, following the NICE guidance for healthcare evaluations [24], comparing differential mean costs and QALYs between the trial arms with usual care as the reference group (acupuncture vs. usual GP care and Alexander lessons vs. usual GP care). In each case an incremental cost-effectiveness ratio (ICER) was calculated using the mean difference in cost between two trial arms divided by the mean difference in effectiveness (QALYs in the primary analysis). Differential mean QALYs were adjusted for baseline EQ-5D score and differential mean costs were adjusted for baseline costs using seemingly unrelated regression models. The analysis was based on complete cases, whereby results were analysed only for participants who had both cost and outcome data; i.e. complete data on healthcare resource use at baseline, 6 and 12 months (NHS appointments and prescription medication), intervention costs and EQ-5D at baseline, 6 and 12 months (in order to calculate QALYs). Data were treated as missing if participants did not return their questionnaire at 6 or 12 months. In addition, for those who returned their questionnaire, total prescriptions were coded as missing if the participant answered ‘yes’ to taking prescription medication but all questions about prescription medications were left blank at 6 or 12 months. Finally, data on total NHS appointments were coded as missing if all questions about appointments were left blank at 6 or 12 months.

Analyses were conducted in Stata 13.1. Differences in resource use and cost were tested for significance using independent sample *t* tests.

Cost data were bootstrapped to account for the skewness that is generally observed (as costs cannot be negative, and there are often some individuals with much higher resource use than average). Bootstrapping resampled the data using 1000 replications. Confidence intervals for differential costs and QALYs were estimated using bias corrected and accelerated confidence intervals (BCA) [34]. All analyses were adjusted for GP practice size.

In order to confirm the validity of the base case analysis, a series of sensitivity analyses were completed to test the robustness of the original estimate. For analyses using cost per QALY: (i) broader societal costs arising from private healthcare expenditure and productivity losses were included; (ii) healthcare resources not relating to neck pain were excluded; (iii) missing data on EQ-5D and costs were imputed. Finally, an analysis using NPQ as the outcome, rather than EQ-5D was also conducted.

Multiple imputation was used to impute missing data items for participants who had missing data on costs and QALYs, with data assumed to be missing at random. All missing values were imputed using Rubin’s method, by means of iterative chain equations, using variables important in the effectiveness analysis (NPQ scores, duration of neck pain, age, gender, and city), health resource use costs (log transformed), baseline EQ-5D score, treatment allocation and perceived stress, which significantly predicted missing data [35–39]. Following imputation, the results of two participants who died during the course of the trial were coded as missing.

## Results

The effectiveness findings of the ATLAS trial indicated clinically relevant reductions in neck pain NPQ scores at 12 months for both acupuncture (32%) and Alexander lessons (31%), compared to usual care (23%); adjusted differences of 3.92 and 3.79 percentage points, respectively (n = 517)[14]. For the primary economic analysis, full economic data (complete data on costs and outcomes at all time points) were available for 293 participants: 104 acupuncture, 89 Alexander and 100 usual care participants, representing 58% of the 509 participants at baseline (N = 509 differs from the analytic sample of 517 for the clinical effectiveness analysis [14] as, per-protocol, we excluded 8 participants randomised in error). Although responders (participants with full economic data) and non-responders (participants with missing data) were similar demographically, responders had significantly lower NPQ scores at all time points, greater improvement in neck pain, higher EQ-5D scores at all time points and consumed fewer healthcare resources, compared to non-responders (see S1 Table for more information).

### Outcomes

EQ-5D scores at baseline, 6 and 12 months are presented in Table 2. QALY values improved in all three groups, but more so for both interventions. Once adjusted for baseline EQ-5D, incremental QALY gains were slightly greater at 12 months in the acupuncture group compared to usual care at +0.032 (95% CI: 0.0001 to 0.062) than in the Alexander group at +0.025 (95% CI: −0.007 to 0.058).

**Table 2:** Change in outcomes between intervention groups.

	Acupuncture			Alexander lessons			Usual care	
	Mean (SD)	[Min-max]	Statistical significance (p-value)*	Mean (SD)	[Min-max]	Statistical significance (p-value)*	Mean (SD)	[Min-max]
*EQ-5D utilities*^*a*^								
Baseline	0.683 (0.179)	[-0.016 to 1]	0.60	0.698 (0.195)	[-0.239 to 1]	0.95	0.697 (0.179)	[-0.074 to 1]
6 months	0.755 (0.190)	[-0.016 to 1]	0.20	0.757 (0.162)	[-0.008 to 1]	0.17	0.719 (0.214)	[-0.055 to 1]
1 year	0.766 (0.188)	[-0.016 to 1]	0.15	0.763 (0.197)	[-0.008 to 1]	0.22	0.727 (0.197)	[-0.016 to 1]
*QALY over 1 year*								
Unadjusted differential QALY^b^	0.740 (0.159)	[0.0445 to 1]	0.28	0.744 (0.145)	[0.166 to 1]	0.22	0.715 (0.169)	[0.0215 to 1]
Adjusted differential QALY	0.025			0.029				
(bootstrapped 95% CI)^c^	0.032 (0.001 to 0.062)			0.025 (-0.007 to 0.058)				
*NPQ percent score*^*d*^								
Baseline	38.16 (7.80)	[27.78–59.38]	0.84	36.87 (8.18)	[27.78–66.67]	0.21	38.41 (8.75)	[27.78–66.67]
6 months	25.10 (13.47)	[0–56.25]	<0.001	24.16 (13.59)	[0–69.44]	<0.001	32.05 (12.59)	[0–63.89]
1 year	25.25 (14.57)	[0–63.89]	<0.05	23.84 (14.22)	[0–62.50]	<0.05	29.84 (14.22)	[2.78–66.67]
*Change in NPQ percent score*								
Change in NPQ score	-33.64 (37.73)	[-100 to 109.09]	<0.05	-36.37 (33.82)	[-100 to 40.00]	<0.05	-22.69 (32.42)	[-92.31 to 60.00]
Unadjusted differential NPQ score^b^	-10.96			-13.68				
Adjusted differential NPQ scores (bootstrapped 95% CI)^e^	-10.58 (-19.67 to -1.35)			-12.79 (-22.07 to -4.12)				

*p-values from independent samples t-test comparing means for acupuncture vs. usual care and Alexander vs. usual care

^a^ Base case analysis: N = 293

^b^Differential QALYs or NPQ scores calculated as mean scores of acupuncture group minus mean scores of usual care group and mean scores of Alexander group minus mean scores of usual care group.

^c^Adjusted for baseline EQ-5D index score and practice size. 95% non-parametric bias-corrected confidence intervals based on 1,000 bootstrap replications.

^d^ Sensitivity analysis: N = 298

^e^Adjusted for baseline NPQ score and practice size. 95% non-parametric bias-corrected confidence intervals based on 1,000 bootstrap replications.

CI, confidence interval.

NPQ scores at baseline, 6 and 12 months are presented in Table 2, as well as percent change over 12 months, among 298 participants with full cost and NPQ data at all time points. NPQ scores decreased, indicating an improvement in neck pain, in all three arms by 6 and 12 months, but significantly greater improvements were made in the acupuncture (% change -33.64) and Alexander groups (% change -36.37) compared to the usual care group (% change -22.69).

### Resource use and costs

Table 3 presents mean NHS healthcare resource use, private healthcare resource use and productivity losses (both trial and non-trial related) over the 12 month trial period. There were no significant differences between usual care and either acupuncture or Alexander Technique arms in terms of NHS appointments or prescription items. Significantly more people paid for acupuncture in the acupuncture arm, and the same was true for Alexander lessons in the Alexander arm, mainly representing people who attended all trial sessions, and paid for extra. There were no significant differences between usual care and either acupuncture or Alexander Technique arms in terms of other private appointments, or days off for neck pain.

**Table 3:** Resource utilisation over 12 month follow-up by treatment group.

	Acupuncture	Alexander lessons	Usual care
	(n = 104) Mean (SD)	(n = 89) Mean (SD)	(n = 100) Mean (SD)
**NHS Healthcare resource use**			
*NHS appointments*^*a*^			
General Practitioner appointments	3.25 (2.94)	4.01 (3.94)	3.59 (3.43)
Practice Nurse appointments	1.22 (1.47)	1.44 (2.50)	1.06 (1.38)
Physiotherapist visits	1.01 (2.56)	1.43 (2.96)	1.12 (2.53)
Hospital outpatient visits	0.85 (1.94)	0.93 (1.75)	0.77 (1.81)
Accident and Emergency admissions	0.23 (0.69)	0.09 (0.44)	0.09 (0.35)
Hospital day case admissions	0.13 (0.40)	0.18 (0.65)	0.09 (0.32)
Other hospital admissions	0.05 (0.26)	0.02 (0.21)	0.03 (0.17)
*Prescription medication*^*b*^			
Prescription items (all)	11.42 (18.74)	15.60 (22.17)	11.64 (17.92)
Prescription items for neck pain	1.84 (5.60)	2.70 (5.53)	3.83 (9.15)
**Private healthcare for neck pain**^c^			
Additional acupuncture sessions	1.51 (3.91)**	0.20 (1.21)	0.14 (1.07)
Additional Alexander Technique lessons	0	0.54 (2.01)*	0
Other private appointments for neck pain^d^	0.86 (3.06)	0.98 (3.40)	2.11 (6.44)
**Productivity losses**			
Days off work due to neck pain^e^	0.38 (1.99)	1.44 (7.51)	2.27 (11.33)
Hours taken to attend sessions (for those in full time work)^f^	19.68 (5.43)	25.91 (9.26)	-

*p<0.05

**p<0.001 (independent samples t-test comparing means for acupuncture vs. usual care and Alexander vs. usual care). Neck pain prescriptions t-test comparing usual care and acupuncture borderline significance (p = 0.06).

^**a**^Total NHS appointments coded as missing if all questions about NHS appointments were left blank at 6 or 12 months.

^**b**^Total prescriptions coded as missing if the participant answered ‘yes’ to taking prescription medication but all questions about prescription medications were left blank at 6 or 12 months.

^c^Private healthcare coded as missing if responded ‘yes’ to paying for additional treatments, but left information about the number of treatments blank.

^d^Other private treatment reported included: yoga, massage therapy, physiotherapy, chiropractor and osteopath appointments.

^e^Excluding time off work to attend intervention sessions.

^f^N = 42 for acupuncture, N = 33 for Alexander lessons.

SD, Standard deviation

Table 4 presents NHS healthcare costs and societal costs during the trial. After adjusting for baseline costs, the average incremental NHS healthcare costs were £451 higher for acupuncture compared to usual care and £667 higher in the Alexander arm and average societal costs were £509 and £862 higher in the acupuncture and Alexander arms, respectively, compared to usual care. Despite somewhat higher resource use from both a healthcare and a societal perspective in the intervention arms compared to usual care (not statistically significant), higher costs for both acupuncture and Alexander lessons were largely attributable to the costs of providing the interventions, at an average of £389 per participant for acupuncture and £587 for Alexander lessons.

**Table 4:** Intervention, NHS healthcare and societal costs (£) over 12 month follow-up between intervention groups: Complete case analysis.

	Acupuncture	Alexander lessons	Usual care
	(n = 104)	(n = 89)	(n = 100)
**Intervention costs**			
Mean (95% CI)	389.38 (372.68–406.07)	586.96 (549.08–624.83)**	-
Min-max	[0–420]	[0–660]	
**Healthcare resource use costs**			
Mean (95% CI)	558.01 (412.73–703.28)	612.72 (415.27–810.17)	484.27 (370.78–597.75)
**Private healthcare for neck pain**			
Mean (95% CI)	85.13 (51.46–118.79)	69.16 (22.19–116.13)	60.25 (28.00–92.51)
**Lost productivity costs**^**a**^			
Mean (95% CI)	185.61 (74.46–296.75)	465.27 (61.95–868.58)	176.34 (50.39–302.29)
**Total NHS healthcare costs**			
Mean (95% CI)	947.38 (800.43–1094.33)**	1199.68 (999.84–1399.51)**	484.27 (370.78–597.75)
**Incremental NHS healthcare costs**^**b**^			
Unadjusted difference	463.12	715.41	-
Adjusted difference (bootstrapped 95% CI)^c^	451.32 (285.29 to 634.83)	667.24 (472.28 to 896.42)	
**Total societal costs**			
Mean (95% CI)	1218.11 (1036.85–1399.38)**	1734.10 (1268.44–2199.76)**	720.86 (530.61–911.10)
**Incremental societal costs**^**b**^			
Unadjusted difference	497.25	1013.24	-
Adjusted difference (bootstrapped 95% CI)^d^	509.44 (252.11 to 775.93)	861.70 (491.52 to 1286.13)	

**p<0.001 (independent samples t-test comparing means for acupuncture vs. usual care and Alexander vs. usual care, or acupuncture vs. Alexander for intervention costs)

^a^Includes time off work due to neck pain and time off work to attend intervention sessions

^b^Incremental costs calculated as mean costs of acupuncture group minus mean costs of usual care group and mean costs of Alexander group minus mean costs of usual care group.

^c^Adjusted for baseline NHS healthcare costs and practice size. 95% non-parametric bias-corrected confidence intervals based on 1,000 bootstrap replications.

^d^Adjusted for baseline NHS societal costs and practice size. 95% non-parametric bias-corrected confidence intervals based on 1,000 bootstrap replications.

### Cost effectiveness: Base case analysis

**Fig 1:**
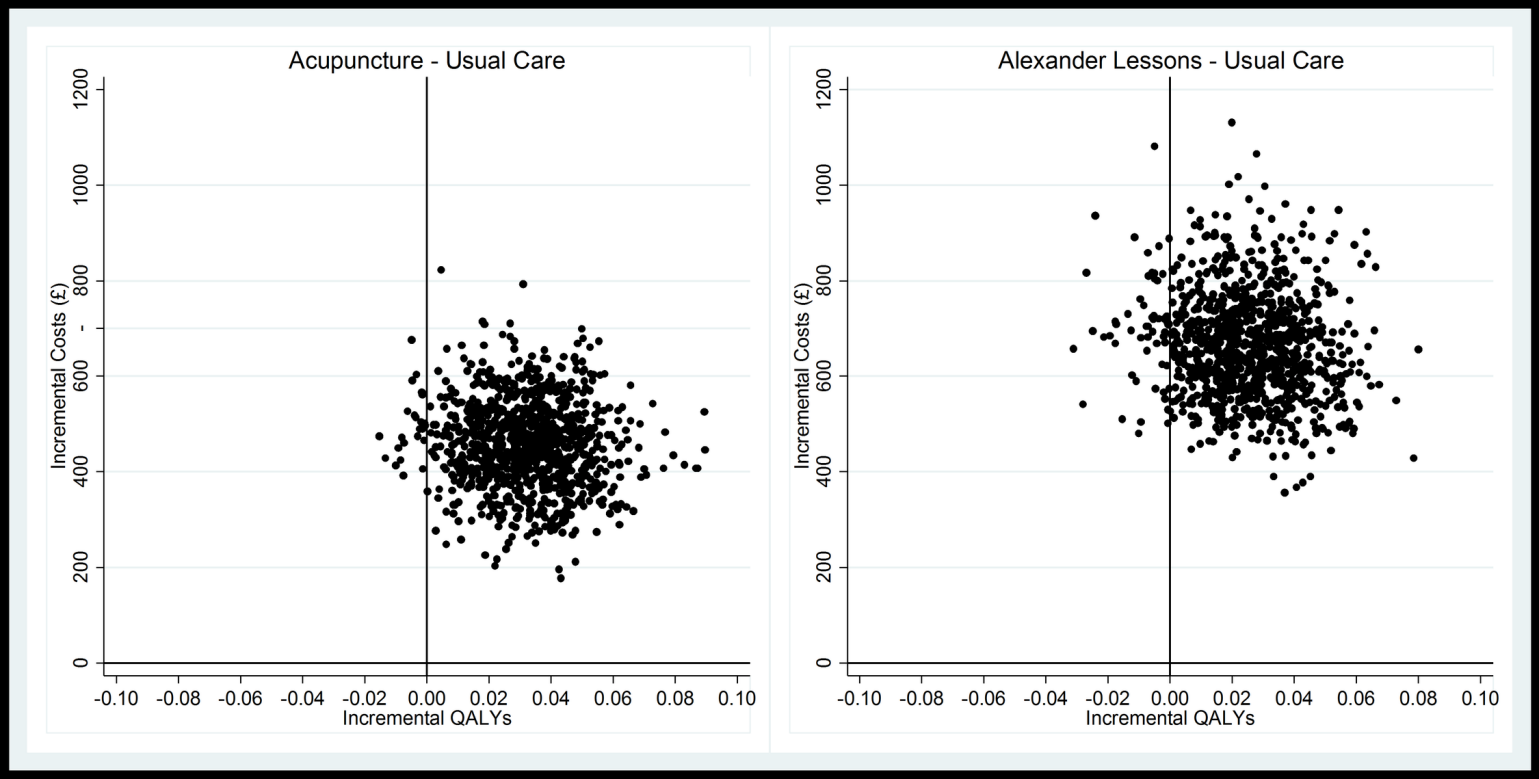
Cost effectiveness planes for the management of neck pain from a healthcare perspective (base case analysis). Planes show incremental costs and QALYs based on 1000 bootstrap cost-effect pairs (adjusted for baseline EQ-5D, baseline healthcare costs and practice size).

**Fig 2:**
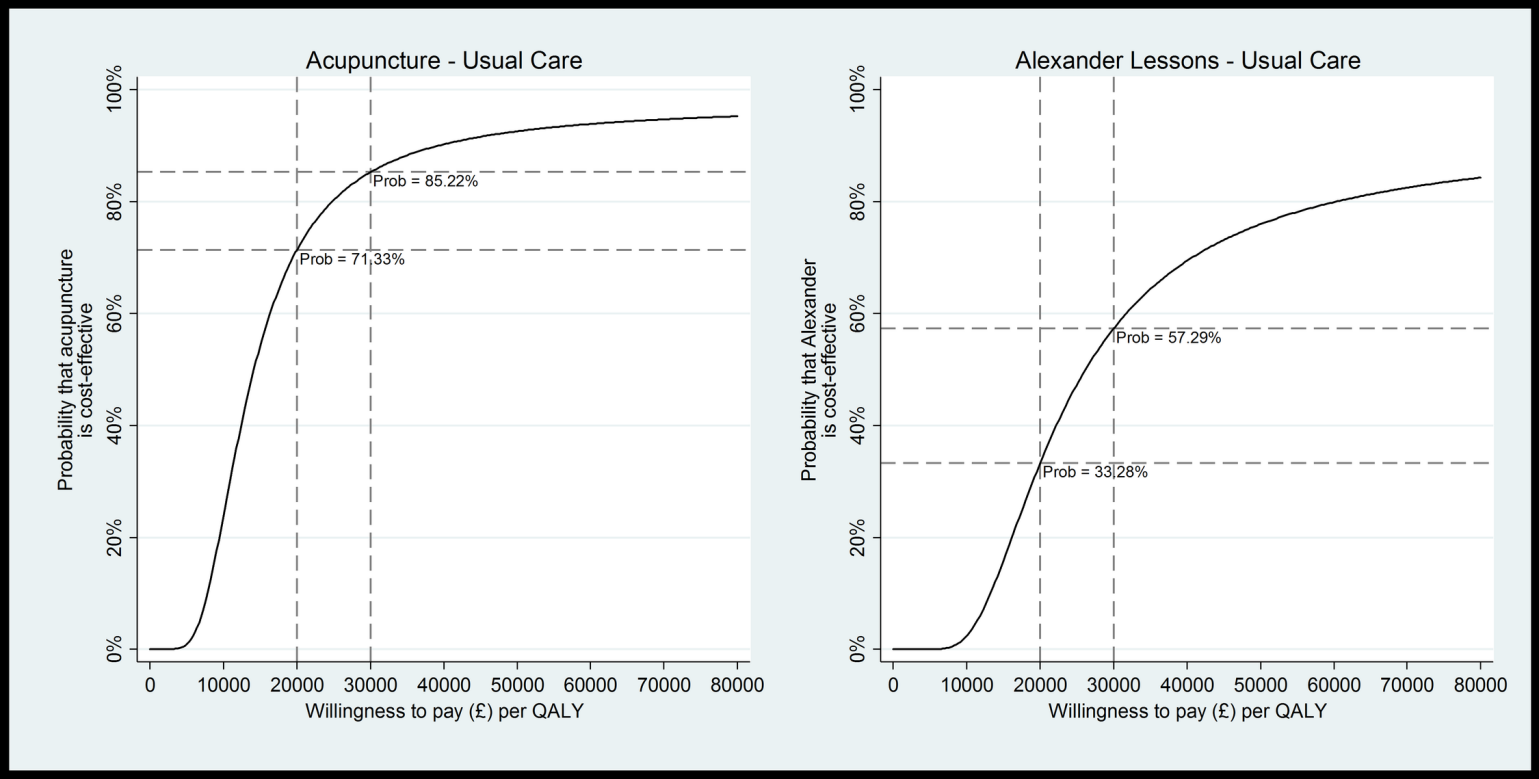
Cost effectiveness acceptability curves (CEACs) for the management of neck pain from a healthcare perspective (base case analysis). Graphs show cost per QALY gained based on 1000 bootstrap cost-effect pairs (adjusted for baseline EQ-5D, baseline healthcare costs and practice size).

The primary base case analysis generated ICERs of £18,767 per QALY gained for acupuncture and £25,101 per QALY for Alexander lessons (Table 5). Fig 1 shows the uncertainty around the cost-effect pairs, with comparatively less uncertainty around effectiveness for acupuncture than for Alexander lessons, demonstrated also by the wide confidence intervals around the point estimate for the Alexander arm. Given that NICE are willing to pay £20,000-£30,000 per QALY gained, Fig 2 shows that, in this scenario acupuncture is likely to be cost effective, with a probability of 71% at £20,000 and 85% at £30,000 per QALY. Alexander lessons are unlikely to be cost effective at £20,000 per QALY gained (33% probability), but may be cost effective at £30,000 (57% probability).

**Table 5:** Sensitivity analyses (QALYs).

Analysis	Acupuncture vs. usual care				Alexander lessons vs. usual care		
	Sample size	Incremental cost (£)	QALYs gained	ICER (£)	Incremental cost (£)	QALYs gained	ICER (£)
Base case	293	451.32^a^	0.032^b^	18,767/QALY	667.24^a^	0.025^b^	25,101/QALY
		(285.29 to 634.83)	(0.001 to 0.062)	(4,426 to 74,562)^c^	(472.28 to 896.42)	(-0.007 to 0.058)	(-150,208 to 248,697)^c^
Inclusion of societal costs	293	509.44^d^	0.032^b^	20,151/QALY	861.70 ^d^	0.025^b^	35,552/QALY
		(252.11 to 775.93)	(0.001 to 0.062)	(3,659 to 86,635)^e^	(491.52 to 1286.13)	(-0.007 to 0.058)	(-172,253 to 329,091)^e^
Including only healthcare costs relating to neck pain	293	375.46^a^	0.032^b^	15,364/QALY	576.81^a^	0.025^b^	20,065/QALY
		(328.58 to 425.78)	(0.001 to 0.062)	(4,156 to 56,763)^c^	(522.85 to 627.61)	(-0.007 to 0.058)	(-112,735 to 241,192)^c^
Imputation of QALYs and cost	507^f^	690.02^a^	0.019^b^	43,838/QALY	884.41^a^	0.010^b^	121,269/QALY
		(516.39 to 894.26)	(-0.005 to 0.044)	(-216,427 to 395,047)^c^	(727.87 to 1059.61)	(-0.014 to 0.034)	(-854,671 to 1,014,592)^c^

^a^Adjusted for baseline NHS healthcare costs and practice size. 95% non-parametric bias-corrected confidence intervals based on 1,000 bootstrap replications.

^b^ Adjusted for baseline EQ-5D index score and practice size. 95% non-parametric bias-corrected confidence intervals based on 1,000 bootstrap replications.

^c^Based on 1000 bootstrap cost-effect pairs. Adjusted for baseline EQ-5D, baseline healthcare costs and practice size

^d^Adjusted for baseline societal healthcare costs and practice size. 95% non-parametric bias-corrected confidence intervals based on 1,000 bootstrap replications.

^e^ Based on 1000 bootstrap cost-effect pairs. Adjusted for baseline EQ-5D, baseline societal costs and practice size

^f^Excluding 2 participants who died

### Sensitivity analyses

Table 5 presents the cost-effectiveness results of the sensitivity analyses. Taking a broader societal perspective resulted in reduced cost-effectiveness (ICERs of £20,151 and £35,552 per QALY gained for the acupuncture and Alexander arms, respectively). Excluding healthcare resource use not directly relating to neck pain improved cost-effectiveness somewhat for both interventions (£15,364 per QALY gained for acupuncture and £20,065 per QALY for Alexander lessons), however, this analysis should be treated with some caution due to missing data (see discussion).

Multiple imputation was used to impute missing data on costs and QALYs. The S1 Table presents a comparison of the characteristics of participants with complete data (CCA analysis), and those with missing data, who were excluded from the primary CCA, by trial arm. Overall, across all trial groups, participants with complete data reported lower levels of neck pain (although they did have a longer prior duration of neck pain), had greater improvements in neck pain and QALYs during the trial, and consumed fewer healthcare resources, compared to participants with missing data. Around 40% of participants had some missing data in the acupuncture and usual care arms, compared to 48% in the Alexander group. Unlike the CCA analysis, participants with missing data in the acupuncture and Alexander arms did not make significant improvements in neck pain scores compared to usual care, and QALY gains were actually greatest in the usual care arm. Imputation resulted in higher costs in the intervention groups compared to the base case analysis, as well as smaller incremental QALY gains, resulting in both interventions exceeding the NICE willingness to pay thresholds, with ICERs of £43,838 per QALY gained for acupuncture and £121,269 per QALY for Alexander lessons. These ICERs were associated with high levels of statistical uncertainty as the small, non-significant changes in QALYs resulted in wide confidence intervals around the point estimates for both interventions.

Using NPQ score, both Alexander lessons and Acupuncture were more expensive than usual care, but also more effective, as in the primary analysis. This generated an ICER of £42.21 per 1 percentage point reduction in NPQ score for acupuncture and £54.49 per 1 percentage point reduction for Alexander lessons over 12 months (see S2 Table for more information).

## Discussion

To our knowledge this is the first full economic evaluation conducted within a randomised controlled trial to assess the Alexander Technique lessons for people with chronic neck pain, and the first economic evaluation of acupuncture over the longer term, with follow-up after 12 months for interventions typically delivered in 5 months. In the primary analysis, QALY gains were found to be associated with both interventions, indicating a modest health benefit compared to usual care. Incremental NHS healthcare costs were £451 and £667 higher in the acupuncture and Alexander arms, respectively, compared to usual GP care. The main cost driver was the cost to provide the interventions, due to their time-intensive nature, with NHS resource use differences non-significant between groups. Acupuncture is likely to be cost-effective, with a probability of 71% at NICE’s lower willingness to pay threshold of £20,000 per QALY, increasing to 85% at £30,000. Due to the higher cost, coupled with a slightly lower QALY gain, Alexander lessons are unlikely to be cost effective (33% probability at £20,000), but may be cost effective if decision makers are willing to pay the higher threshold value of £30,000 per QALY gained, with a probability of 57%.

Multiple sensitivity analyses were conducted, with the conclusions of the primary analysis shown to be generally robust. When healthcare resource use due to problems other than neck pain were excluded, an approach recommended by NICE [24], acupuncture was comfortably within the lower NICE threshold for willingness to pay of £20,000 per QALY (ICER £15, 364) and Alexander lessons were on the threshold (£20,065), but again with considerable uncertainty reflected by extremely wide confidence intervals. Because it is difficult for participants to assess the amount of their healthcare resource use that is directly related to the disease of interest, the validity of basing cost effectiveness analyses on such assessments is an area of current debate and research [40]. In order to address this, patients were asked to report all healthcare resource use, and to additionally indicate if they thought the use was due to neck pain. Unfortunately, this method resulted in additional participant burden in an already lengthy self-report questionnaire and likely contributed to a large amount of missing data for healthcare resource use in the trial. Furthermore, asking participants to report the number of appointments for both neck pain, and overall appeared to have caused some confusion, and pre-specified assumptions had to be made in many cases regarding the responses given (see S1 Appendix).

When societal costs rather than NHS costs are considered, the probability that acupuncture or Alexander Technique lessons are cost-effective was somewhat reduced. In a previous study, including productivity losses resulted in improved cost-effectiveness of acupuncture compared to usual care [26], however, in our study there were only moderate non-significant differences in time off work for neck pain between groups, and we also included the time taken to attend intervention sessions, resulting in higher productivity losses in both intervention groups, but particularly for the Alexander group due to the higher number of sessions.

Levels of missing data were relatively high in the trial, with only 58% participants having complete data for the health economic evaluation (complete data on costs and outcomes at all time points as detailed in the Methods). This level of missing data is not unusual in studies of this type, particularly due to the length of follow-up and the need for complete data on costs and outcomes at all time points, and is in fact similar to the level of missing data in a previous acupuncture trial [26]. The concern with missing data is that the data will not be missing at random, which may introduce bias. Comparisons revealed that participants with complete data, although similar demographically, could generally be described as healthier and had lower healthcare resource use costs than participants with missing data. Moreover, in the between-group comparisons, participants with missing data from the two intervention groups had smaller improvements in neck pain and quality of life compared to participants with missing data in the usual care arm. After imputing missing data, neither intervention remained cost-effective, albeit with a high level of statistical uncertainty. Based on the data available, imputation provides our best prediction of missing values, with the inherent uncertainty that comes with using predictive methods.

Finally, analyses using neck pain and associated disability, rather than QALY were also conducted due to the relative insensitivity of the EQ-5D compared to the specific NPQ score. Although there are no accepted willingness to pay thresholds for an improvement in NPQ score, results of the analysis were largely in line with the base case–indicating that both acupuncture and Alexander lessons conferred a greater but more costly health benefit compared to usual care, with Alexander lessons being more costly than acupuncture but slightly more effective.

There is scant literature examining the cost-effectiveness of either acupuncture or Alexander lessons for chronic neck pain, with which to make direct comparison. However, multiple previous studies deemed to be at low risk of bias have reported similar QALY gains for acupuncture compared to controls, with consistently cost-effective results (cost per QALY <£20,000) for a range of chronic pain problems [11], including one large German study comparing acupuncture to usual care for chronic neck pain over a three month period [41]. A systematic review of manual or exercise therapies found some moderate evidence of cost savings with these therapies for neck pain, but none of the included studies used Alexander Technique lessons [42]. We are aware of one previous cost-utility analysis comparing 6 Alexander Technique lessons to usual care for chronic back pain in the UK, which found Alexander lessons to be cost-effective over a 12 month period [21]. However, this represents a less intensive (and therefore less costly) intervention than in our study. The authors did also present analyses for 24 lessons, but their comparison was to the 6 lesson alternative, rather than to usual care. Furthermore, the study was hampered by similar issues with missing economic data, particularly for QALYs.

Overall, the results of the primary base case economic evaluation suggest that acupuncture and Alexander Technique lessons both confer a greater health benefit compared to usual care. At a cost of around £389 per person to provide, and with only a moderate increase in healthcare resource use, acupuncture is likely to be considered cost-effective compared to usual care. However, the more time-intensive and therefore more expensive Alexander Technique lessons (at around £587 per person on average) are less likely to be considered cost-effective, given current willingness to pay thresholds and the considerable statistical uncertainty, reflected by a very wide confidence intervals. Nevertheless, in cases where acupuncture is not an option (e.g. for a needle phobic patient), Alexander Technique lessons may be considered. Multiple sensitivity analyses generally suggested that the results of the primary analysis were robust. However, the main weakness of our analysis was the large amount of missing resource use data. The result of our imputed analysis indicated acupuncture was no longer cost effective at accepted thresholds. However, due to the level of missing data, the analysis also introduced a great deal of uncertainty, with wide confidence limits around the estimate. Nevertheless, given that complete case analyses can produce biased results, we suggest cautious interpretation of our initial estimates, which may be reflective of a healthier sub-population. Moreover, in the smaller sub-group of participants with missing data smaller improvements in health outcomes in the intervention groups than in the usual care group, introducing another possible level of bias to the complete case analysis. Further research is needed to explore the long term cost-effectiveness of acupuncture and Alexander Technique lessons for chronic neck pain patients, whilst addressing issues with data completeness that are commonplace in evaluations of this kind.

## Supporting information

S1 Table Characteristics of participants included and excluded from the CCA analysis, by randomised group. (DOCX)

S2 Table Incremental cost effectiveness analysis: NPQ scores (N = 298). (DOCX)

S1 Appendix Pre-specified assumptions for coding overall and neck-pain specific resource use. (DOCX)

S1 File ATLAS study protocol. (PDF)
