# Supplementary material for: An economic evaluation of Alexander Technique lessons or acupuncture sessions for patients with chronic neck pain: A randomized trial (ATLAS)
Source: PLoS One. 2017 Dec 6;12(12):e0178918. doi: 10.1371/journal.pone.0178918 (PMC5718562; doi:10.1371/journal.pone.0178918)
Supplement: S1 Table — (DOCX) [file pone.0178918.s001.docx]

Table S1: Characteristics of participants included and excluded from the CCA analysis, by randomised group

| **Variable** | **Acupuncture** | | **Alexander Technique lessons** | | **Usual care** | |
| --- | --- | --- | --- | --- | --- | --- |
|  | **N** | **Mean (95% CI)**  **or**  **N (%)** | **N** | **Mean (95% CI)**  **or**  **N (%)** | **N** | **Mean (95% CI)**  **or**  **N (%)** |
| **Total trial at baseline**  **CCA**  **Excluded** |  | 172  104 (60.5%)  68 (39.5%) |  | 171  89 (52.0%)  82 (48.0%) |  | 166  100 (60.2%)  66 (39.8%) |
| **Age**  CCA  Excluded  **Gender (Female)**  CCA  Excluded  **Education (years)**  CCA  Excluded  **Duration of neck pain (months)**  CCA  Excluded  **NPQ percent score^a^**  *Baseline*  CCA  Excluded  *6 months*  CCA  Excluded  *1 year*  CCA  Excluded  **Change in NPQ percent score**  CCA  Excluded  **EQ-5D utilities**  *Baseline*  CCA  Excluded  *6 months*  CCA  Excluded  *1 year*  CCA  Excluded  **QALY over 1 year**  CCA  Excluded  **NHS resource use (£)**  *Baseline*  CCA  Excluded  *6 months*  CCA  Excluded  *1 year*  CCA  Excluded | 104  68  104  68  99  59  104  68  104  68  104  51  104  45  104  45  104  67  104  49  104  44  104  39  104  46  104  24  104  25 | 52.47 (49.90 to 55.04)  51.37 (47.81 to 54.93)  74 (71.15)  45 (66.18)  18.31 (17.27 to 19.35)  17.75 (16.80 to 18.69)  112.42 (90.50 to 134.34)  91.54 (67.07 to 116.02)*  38.24 (36.72 to 39.75)  41.85 (38.9 to 44.71)  25.23 (22.62 to 27.85)**  30.36 (26.06 to 34.65)  25.31 (22.47 to 28.15)*  29.65 (24.48 to 34.81)  -33.62 (-41.00 to 26.25)*  -29.08 (-39.07 to 19.09)  0.683 (0.648 to 0.718)  0.593 (0.524 to 0.662)  0.755 (0.719 to 0.792)  0.657 (0.586 to 0.728)  0.766 (0.729 to 0.802)  0.665 (0.575 to 0.755)  0.740 (0.709 to 0.771)  0.651 (0.570 to 0.732)  357.03 (236.21 to 477.84)  561.89 (296.48 to 827.30)  211.69 (153.05 to 270.34)  512.17 (193.73 to 830.61)  346.31 (221.21 to 471.42)  496.02 (27.68 to 964.35) | 89  82  89  82  87  76  89  82  89  82  89  53  89  55  89  55  89  81  89  50  89  53  89  43  89  60  89  20  89  32 | 54.71 (51.67 to 57.74)  52.36 (49.10 to 55.62)  62 (69.66)  58 (70.73)  18.69 (17.29 to 20.09)  17.64 (16.36 to 18.93)  122.76 (99.10 to 146.43)  88.83 (69.52 to 108.14)*  36.77 (35.04 to 38.51)  42.42 (39.27 to 45.56)  24.31 (21.41 to 27.20)**  32.06 (27.21 to 36.90)  23.83 (20.86 to 26.79)*  32.58 (27.90 to 37.27)  -36.26 (-43.05 to 29.46)*  -23.33 (-32.80 to 13.86)  0.698 (0.657 to 0.739)  0.595 (0.538 to 0.652)  0.757 (0.723 to 0.791)  0.629 (0.564 to 0.694)  0.763 (0.721 to 0.804)  0.645 (0.572 to 0.717)  0.744 (0.713 to 0.774)  0.611 (0.548 to 0.675)  393.85 (286.36 to 501.33)  430.26 (274.74 to 585.78)  297.00 (186.33 to 407.68)  302.28 (104.13 to 500.43)  315.71 (205.77 to 425.66)  415.10 (233.67 to 596.54) | 100  66  100  66  94  64  100  65  100  66  100  45  100  40  100  40  100  66  100  44  100  39  100  35  100  49  100  15  100  21 | 53.69 (51.12 to 56.26)  53.93 (50.66 to 57.20)  69 (69.00)  47 (71.21)  18.51 (17.62 to 19.41)  18.64 (16.65 to 20.63)  117.54 (97.47 to 137.61)  135.63 (106.94 to 164.32)  38.42 (36.67 to 40.16)  42.92 (39.57 to 46.28)  32.00 (29.49 to 34.50)  35.30 (30.23 to 40.38)  29.76 (-29.37 to 16.47)  33.95 (29.03 to 38.86)  -22.92 (-29.37 to 16.47)  -19.85 (-29.12 to 10.57)  0.697 (0.661 to 0.732)  0.590 (0.515 to 0.665)  0.719 (0.676 to 0.761)  0.643 (0.556 to 0.731)  0.727 (0.688 to 0.766)  0.685 (0.613 to 0.758)  0.715 (0.682 to 0.749)  0.665 (0.592 to 0.739)  299.31 (226.65 to 371.97)  477.65 (213.81 to 741.49)  232.05 (160.46 to 303.63)  198.26 (97.14 to 299.38)  252.22 (181.11 to 323.33)  631.77 (160.52 to 1103.02) |

*p<0.05 **p<0.001 (independent samples t-test comparing means for acupuncture vs. usual care and Alexander lessons vs. usual care).
