# Supplementary material for: An economic evaluation of Alexander Technique lessons or acupuncture sessions for patients with chronic neck pain: A randomized trial (ATLAS)
Source: PLoS One. 2017 Dec 6;12(12):e0178918. doi: 10.1371/journal.pone.0178918 (PMC5718562; doi:10.1371/journal.pone.0178918)
Supplement: S2 Table — (DOCX) [file pone.0178918.s002.docx]

Table S2: Incremental cost effectiveness analysis: NPQ scores (N=298)

| **Acupuncture vs. usual care** | | | **Alexander lessons vs. usual care** | | |
| --- | --- | --- | --- | --- | --- |
| **Incremental cost (£)** | **Neck pain score change** | **ICER (£)** | **Incremental cost (£)** | **Neck pain score change** | **ICER (£)** |
| 450.29  (289.05 to 633.74) | -10.58  (-19.67 to -1.35) | 42.21/1% reduction in NPQ score  (16.35 to 217.88) | 691.82  (492.06 to 920.53) | -12.79  (-22.07 to -4.12) | 54.49/1% reduction in NPQ score  (27.71 to 171.82) |

^a^Difference in mean costs/difference in mean QALYs.

^b^Based on 1000 bootstrap cost-effect pairs. Adjusted for baseline EQ-5D, baseline health care costs and practice size
