# Supplementary material for: An economic evaluation of Alexander Technique lessons or acupuncture sessions for patients with chronic neck pain: A randomized trial (ATLAS)
Source: PLoS One. 2017 Dec 6;12(12):e0178918. doi: 10.1371/journal.pone.0178918 (PMC5718562; doi:10.1371/journal.pone.0178918)
Supplement: S1 Appendix — (DOCX) [file pone.0178918.s003.docx]

**S1 Appendix**

**Pre-specified assumptions for coding overall and neck-pain specific resource use**

The answers for total NHS resource use (General Practitioner, Practice Nurse, Physiotherapist, hospital outpatient, Accident and Emergency, hospital day case or other hospital admission) were compared with the answers for resource use reported to be specifically for neck pain. As well as generally comparing answers, two pre-specified assumptions were made with subsequent coding changes as follows:

- Ensured overall NHS resource use could not be missing if neck pain resource use had a value (and replacing overall resource use with neck pain resource use in these cases). For example, if total number of GP visits was left blank, but number of GP visits for neck pain recorded as 3.
- Ensured overall NHS resource use value could not be smaller than the value for neck pain resource use (and replacing overall resource use with neck pain resource use in these cases). For example, if total number of practice nurse appointments reported as 1 and practice nurse appointments for neck pain recorded as 4.
