# Supplementary material for: An economic evaluation of Alexander Technique lessons or acupuncture sessions for patients with chronic neck pain: A randomized trial (ATLAS)
Source: PLoS One. 2017 Dec 6;12(12):e0178918. doi: 10.1371/journal.pone.0178918 (PMC5718562; doi:10.1371/journal.pone.0178918)
Supplement: S1 File — (PDF) [file pone.0178918.s004.pdf]

MacPherson et al. Trials 2013, 14:209
http://www.trialsjournal.com/content/14/1/209

TRIALS

STUDY PROTOCOL Open Access

Alexander Technique Lessons, Acupuncture Sessions or usual care for patients with chronic neck pain (ATLAS): study protocol for a randomised controlled trial

Hugh MacPherson[1*], Helen E Tilbrook[1], Stewart J Richmond[1], Karl Atkin[1], Kathleen Ballard[2], Martin Bland[1], Janet Eldred[1], Holly N Essex[1], Ann Hopton[1], Harriet Lansdown[1], Usman Muhammad[1], Steve Parrott[1], David Torgerson[1], Aniela Wenham[1], Julia Woodman[1,2] and Ian Watt[3]

Abstract

Background: Chronic neck pain is a common condition in the adult population. More research is needed to evaluate interventions aiming to facilitate beneficial long-term change. We propose to evaluate the effect of Alexander Technique lessons and acupuncture in a rigorously conducted pragmatic trial with an embedded qualitative study.

Methods/Design: We will recruit 500 patients who have been diagnosed with neck pain in primary care, who have continued to experience neck pain for at least three months with 28% minimum cut-off score on the Northwick Park Neck Pain Questionnaire (NPQ). We will exclude patients with serious underlying pathology, prior cervical spine surgery, history of psychosis, rheumatoid arthritis, ankylosing spondylitis, osteoporosis, haemophilia, cancer, HIV or hepatitis, or with alcohol or drug dependency currently or in the last 12 months, or actively pursuing compensation or with pending litigation.

The York Trials Unit will randomly allocate participants using a secure computer-based system. We will use block randomisation with allocation to each intervention arm being unambiguously concealed from anyone who might subvert the randomisation process.

Participants will be randomised in equal proportions to Alexander Technique lessons, acupuncture or usual care alone. Twenty 30-minute Alexander Technique lessons will be provided by teachers registered with the Society of Teachers of the Alexander Technique and twelve 50-minute sessions of acupuncture will be provided by acupuncturists registered with the British Acupuncture Council. All participants will continue to receive usual GP care.

The primary outcome will be the NPQ at 12 months, with the secondary time point at 6 months, and an area-under-curve analysis will include 3, 6 and 12 month time-points. Adverse events will be documented. Potential intervention effect modifiers and mediators to be explored include: self-efficacy, stress management, and the incorporation of practitioner advice about self-care and lifestyle. Qualitative material will be used to address issues of safety, acceptability and factors that impact on longer term outcomes.

Discussion: This study will provide robust evidence on whether there are significant clinical benefits to patients, economic benefits demonstrating value for money, and sufficient levels of acceptability and safety.

Trial registration: Current Controlled Trials ISRCTN15186354

* Correspondence: hugh.macpherson@york.ac.uk
[1]Department of Health Sciences, University of York, York YO10 5DD, UK
Full list of author information is available at the end of the article

BioMed Central

© 2013 MacPherson et al.; licensee BioMed Central Ltd. This is an Open Access article distributed under the terms of the Creative Commons Attribution License (http://creativecommons.org/licenses/by/2.0), which permits unrestricted use, distribution, and reproduction in any medium, provided the original work is properly cited.

Background

The problem to be addressed

Optimal care for uncomplicated chronic neck pain has yet to be established [1] Despite decades of research, little advance has been made in reducing the health and economic burden of chronic neck pain [2]. There is some evidence that exercise, mobilisation and manipulation are beneficial [1], although there are concerns regarding the safety of neck manipulation. While psycho-social factors are associated with increasing chronicity, [3] and improved coping strategies result in better outcomes [4], interventions that facilitate change in illness-related behaviours and habits are needed [2]. Many people with chronic neck pain consult acupuncturists and Alexander Technique teachers, largely outside the NHS, and while these interventions are considered relatively safe, the evidence on health outcomes is as yet inconclusive [1].

The principal research questions to be addressed

Our principal research question asks: What is the clinical and economic impact when comparing acupuncture treatment plus usual care to usual care alone, and Alexander Technique lessons plus usual care to usual care alone in patients with chronic neck pain who are recruited from primary care? We will also compare acupuncture to Alexander Technique lessons to estimate possible clinical differences. We will determine whether putative benefits are clinically worthwhile. We will compare the cost-effectiveness of the alternative strategies, and if these interventions are at an additional cost to usual care, then we will ascertain whether they are worth paying for in terms of cost per Quality Adjusted Life Year (QALY) gained, in the context of the National Institute for Health and Care Excellence (NICE) threshold, which ranges from £20,000 to £30,000 [5]. We will also assess the acceptability and safety profile of acupuncture and Alexander Technique lessons for this patient group. In this study, we have used our pilot trial on acupuncture for chronic neck pain (ISRCTN06223266) [6] to inform the design of the full-scale trial.

How the results of the trial will be used to change patient management

Acupuncture and Alexander Technique lessons are two interventions that are not widely provided within the NHS, yet have the potential to safely deliver longer term benefits for spine-related conditions. Both interventions involve individualised sessions that include support for sustained improvement in health, based on changes in self-care and/or lifestyle [7,8]. Using pragmatic trial designs, where the trial practitioners can provide interventions similar to what they normally would provide in their routine care, long-term benefits have been observed in acupuncture at 12 and 24 months for headache and back pain respectively [9,10]. Acupuncture was found to be cost-effective [11,12]. A Medical Research Council funded trial of Alexander Technique lessons for back pain patients found long-term clinical and cost-effectiveness benefits at 12 months [13,14].

NICE now recommends acupuncture as a referral option within primary care for people with persistent low back pain [15]. The clinical effectiveness and cost-effectiveness illustrated by our York-based trial [11] was central to that decision. The proposed trial has a similar design, and therefore the results will help to inform decision making by policy makers, providers and commissioners as well as patients. If it is found that either of these two interventions is not effective, then decisions to limit the use of these interventions within the NHS will also be in the interests of both the NHS and patients.

How the proposed trial will differ from recently completed trials elsewhere in the United Kingdom or internationally

The proposed trial will complement the two UK-based low back pain trials discussed above, one of acupuncture and one of Alexander Technique lessons [9,13]. A trial conducted in Germany found that acupuncture delivered by physicians in conjunction with routine care was clinically beneficial for chronic neck pain when compared to routine care alone at the end of treatment (that is, 3 months) [16], and also demonstrated cost effectiveness with cost per QALY gained being estimated at €12,469 at 3 months [17]. The proposed trial on chronic neck pain adds an Alexander Technique lesson arm, extends the period of measurement to 12 months, and focuses on a UK-based primary care population. In addition to assessing changes to physical parameters and quality of life, another difference will be our focus on the ways the interventions impact on thoughts/beliefs, self-perceptions, self-efficacy and empowerment, and the potential for these to enhance outcomes over the longer term [7,8].

Methods/Design

Details of the trial

The study is a three-arm pragmatic randomised, controlled trial to evaluate the effectiveness, cost effectiveness, safety and acceptability of acupuncture and Alexander Technique lessons for patients with chronic neck pain. The three arms are: acupuncture plus usual care, Alexander Technique lessons plus usual care, and usual care alone. Participants will be recruited from primary care and randomised to one of the three arms after we have obtained their informed consent to participate in the trial and their completed baseline questionnaire and after they have been screened for eligibility.

This pragmatic design will best answer practical questions regarding the clinical and economic implications of

offering these healthcare options in primary care within the context of existing provision. Our proposal builds on two previous pragmatic trials of acupuncture and Alexander Technique lessons for back pain patients [9,13], both of which were designed to support individualised patient-practitioner interactions and active patient involvement in strategies for longer term change [7,8]. Because both interventions involve active learning (both theoretical and practical/experiential) by the participant, identifying an appropriate and adequate sham is problematic. It is accepted that in addition to the active learning components there are components associated with nonspecific effects (for example, empathy). Yet, even if it were possible to separate out these nonspecific components, there may be an unknown loss of synergy between interacting components [18]. Additionally, in principle, it is difficult to provide scientifically robust sham treatments as controls because the specific mechanisms and causal pathways of acupuncture and Alexander Technique lessons are not known. For example it is unknown whether there are neural pathways associated with both specific and nonspecific effects, such as those associated with self-healing. One resolution to these difficulties is to bypass the question regarding the relative impact of specific and nonspecific components, and to address a different research question, namely asking, 'What is the overall benefit?' For a pragmatic trial to be 'positive', the results must, as a minimum, meet four quantitative challenges: statistical significance, clinical relevance, adequate safety and worthwhile cost-effectiveness. To summarise, the pragmatic design offers best value by providing data that are immediately applicable to patients and providers with real-world comparisons that will assist policy and decision-makers.

We will collect additional data in order to explore:

1) the impact of potential effect modifiers, including preference, belief and expectation, factors often associated with 'placebo' or nonspecific effects
2) the impact of potential effect mediators, including self-efficacy, stress management, and changes in self-care and/or lifestyle, and their association with longer-term outcomes
3) the variation in patient outcome between individual practitioners
4) the identification of sub-groups of patients that respond better/worse to the interventions.

Details of trial interventions

Twenty 30-minute Alexander Technique lessons (600 minutes in total) will be provided by teachers registered with the Society of Teachers of the Alexander Technique (STAT) with at least three years of teaching experience and evidence of a commitment to their continuing professional development. The decision to provide 20 lessons was based on a.) the experience of the ATEAM trial [13], which offered either 6 or 24 lessons and b.) from our consultation with Alexander Technique teachers. Twenty lessons were considered sufficient to consolidate the learning necessary for sustainable change to take place. The 30-minute lesson time was derived from the overall contact time of 600 minutes, equivalent to the acupuncture intervention. The first lesson will be 15 minutes longer to allow the participant to explain his/her problem and to answer questions, and also to allow the teacher to explain what the Alexander Technique is and what will be involved. Subsequent lessons will each contain 30 minutes of active teaching time with the content based on a protocol that was used in the recent ATEAM back pain trial [13] and on further consultation with STAT. Generally lessons will be weekly, with the option of being twice-weekly initially and fortnightly towards the end of the series, and are expected to be completed within five months. The scheduling of appointments will be based on normal practice, involving both the teacher's discretion and the participant's preference. Alexander Technique lessons provide an individualised approach to developing lifelong skills for self-care that help people recognise, understand, and avoid poor habits adversely affecting postural tone and neuromuscular coordination [19]. Lessons incorporate specific principle-driven patient-practitioner interactions designed to empower patients and engage them as active partners in their own recovery [8]. All participating teachers will use both verbal and hands-on instruction in line with the National Occupational Standards Skills for Health guidelines. Teachers will participate in a one-day induction workshop orientated towards the research process and will document components of lessons and adherence in logbooks.

Twelve 50-minute sessions of acupuncture (600 minutes in total) will be provided by professional acupuncturists who practise a style of acupuncture that is based on traditional Chinese medicine (TCM). The decision to provide 12 sessions was based on providing equivalence to the 600 minutes provided by Alexander Technique teachers, given that typically a session lasts 50 minutes. Acupuncturists will be registered with the British Acupuncture Council with at least three years of experience and provide evidence of a commitment to their continuing professional development. The first session will be 15 minutes longer to allow the patient to explain his/her problem and to answer questions. The scheduling of appointments will be based on normal practice, which will involve both the practitioner's discretion and the participant's preference. All sessions are likely to be completed within five months. Acupuncture involves the insertion of fine needles at points indicated by the diagnosis

combined with customised explanations and lifestyle advice that is specific to the acupuncture diagnosis. In this way, acupuncture treatments are provided so as to optimise potential longer term benefits. To support this process, we will use a previously tested acupuncture treatment protocol [6] that incorporates specific theory-driven patient-practitioner interactions designed to engage patients as active partners in their own recovery [7]. All practitioners will participate in a one-day induction workshop (see above). Practitioners will document components of treatment and adherence in logbooks.

Procedures used to identify, shortlist, and recruit Alexander Technique teachers and acupuncturists will be based on those procedures successfully employed in the ACUDep trial, which compared acupuncture, counselling and usual GP care for the treatment of depression (ISRCTN63787732) [20]. As a starting point we shall seek to identify geographical clusters of suitably qualified and experienced Alexander teachers and acupuncturists, who together would be capable of delivering both interventions to trial participants living in Northern England. Each geographical cluster should consist of no less than two Alexander teachers and two acupuncturists, ideally within a two-mile radius. However, additional consideration will be given to local issues relating to public transport and accessibility. Given that patients will be recruited from nearby general practices, most participants will live within walking distance of both an Alexander teacher and acupuncturist. This should serve to minimise any burden on participants in terms of travel, maximise attendance rates, and help to maintain equipoise between both intervention groups. Alexander teachers and acupuncturists who are shortlisted by the research team, and are willing to support the trial, will then be required to submit a curriculum vitae for review, together with any evidence for recent continuing professional development activities. This review process will be conducted in conjunction with relevant professional members of the trial management group. Alexander teachers and acupuncturists who are finally selected will need to provide evidence of professional indemnity insurance and undergo Criminal Records Bureau clearance.

All participants will remain under the care of their GP and will receive usual NHS treatments as well as other care throughout the trial. This is expected to include GP consultations, typically involving brief advice lasting perhaps 10 minutes per consultation plus the offer of prescribed painkillers. Some participants will also be taking over-the-counter medication and/or consult with NHS and non-NHS practitioners. We will record and measure the full range of what constitutes usual care that all participants continue to receive throughout the trial period. This will include additional acupuncture and Alexander Technique lessons, whether paid for out-of-pocket or provided free within the NHS.

Methods for protecting against other sources of bias

Blinding to intervention is not relevant for the proposed trial, which is designed to answer the pragmatic research question that relates to the overall benefit to patients. To estimate the potential impact of the nonspecific components of preference, expectation and belief, we will measure these at baseline and evaluate their impact on outcome, as we have done in previous trials for preference [21] and belief [9]. The York Trials Unit will randomly allocate participants using a secure system ensuring that allocation is unambiguously concealed from anyone who might subvert the randomisation process. We will use block randomisation, which will give us as balanced an allocation as possible within each cluster of participants from each GP practice. Postal questionnaires will be used to collect outcome data after randomisation to ensure no clinician or researcher who knows the allocation can influence the process of data recording, collection and analysis. Intention-to-treat analysis will be used to ensure that the groups will be compared as randomised. Care will be taken to map the components of each intervention in all three arms of the trial, so that we can adequately describe inputs associated with putative differences between group outcomes. Bias due to attrition will be minimised by carefully following up all the participants including drop outs. In order to reduce attrition rates, the final follow-up questionnaire will be accompanied by a £5 incentive to complete it.

Potential risks and hazards to participants and how these are being minimised

Acupuncture and Alexander Technique lessons are relatively safe interventions. However we will involve all practitioners in an induction event prior to their providing sessions for participants within the trial. We will document the procedures designed to minimise the key risks and will induct practitioners into the trial documentation. For acupuncture, the current evidence is that the modality is very safe when practised by competent practitioners. Expected adverse events that have been associated with acupuncture include nausea, fainting, dizziness, sweating, vomiting, bruising, numbness and bleeding at the site of needling, stiffness, headache or migraine, sleeplessness, aggravation of existing symptoms, drowsiness or tiredness after treatment (which might be a concern if participants are driving home), diarrhoea, and emotional reactions, such as anxiety and panic [22,23]. Likewise, for Alexander Technique lessons, the risks are very low as the manual aspects of the lessons involve guidance, not manipulation as generally understood, and are very gentle. Possible adverse events associated with Alexander Technique lessons include transient dizziness during a lesson, tiredness usually beginning 1 to 2 hours or more after a lesson, and muscle

aches (similar to those post-exercise). For the population as a whole, expected hospital admissions might occur due to musculoskeletal injuries, cardiovascular disorders, gastrointestinal disorders, respiratory disorders, cancer-related care, exacerbation of existing medical conditions and elective surgery. We will use the standard operating procedures of the York Trials Unit for monitoring and reporting adverse events. Reporting on serious and non-serious adverse events will involve participants, practitioners and GPs.

Inclusion/exclusion criteria

We will be recruiting typical patients who have consulted their GP for chronic neck pain and are being managed in primary care. Patients will be identified via GP databases, identifying potential participants from relevant READ codes, as adapted from our pilot study [6]. We will also identify some patients through advertisements placed in GP practices. Eligible patients will be aged 18 years and older. They will have had neck pain for at least 3 months, which fits with current thinking on what constitutes 'chronic' neck pain, [1] such that outcome data at this time point will be consistent with that in systematic reviews [24,25]. Given that patients are likely to have disability as well as pain, we will have a 28% minimum cut-off score on the Northwick Park Questionnaire (NPQ), based on the cut-off at 10 points (out of 36), the same as we used in our physiotherapy trial [26]. We will exclude patients with: serious underlying pathology; prior cervical spine surgery; history of psychosis; rheumatoid arthritis ankylosing spondylitis; osteoporosis; haemophilia; cancer; HIV or hepatitis; alcohol or drug dependency currently or in the last 12 months; actively pursuing compensation or with pending litigation; currently receiving acupuncture for neck pain; or having attended one-to-one Alexander Technique lessons in the last 24 months. We will exclude patients at baseline if they are pregnant, not because of risks associated with the interventions, but because of potential loss to follow-up. Participants who become pregnant after entry in to the trial will remain in the trial and continue to attend the intervention. We will exclude patients who have participated in a clinical trial in the previous year if there is potential confounding, or if the burden for the patient appears to be too great. Reasons for exclusions will be documented and reported.

People who are unable to speak or who find it difficult to communicate in English will be excluded from the study. A reasonable level of understanding of English is essential to engage in the sessions with an English-speaking practitioner, to sufficiently understand the practitioners who takes her/his case history, and to understand explanations of the acupuncture treatment or Alexander Technique lessons. No funds have been provided within the research grant to pay for the translation of materials into other languages or to cover costs associated with the use of interpreters, nor does the research team have established access to such services. Therefore while the translation of trial materials into other languages, or the use of interpreters, might enable a small number of patients who do not understand or speak English to take part in the study, in all likelihood this would be neither financially feasible nor practical.

Proposed duration of treatment/intervention period

The trial is designed to be a pragmatic one, whereby practitioners will be encouraged to provide care in a way that, as close as possible, reflects their normal practice. We provided guidance for both the Alexander teachers and the acupuncturists on the number of sessions available within the trial, and the flexibility in delivering these sessions in terms of frequency. Alexander teachers and acupuncturists will be able to discharge participants who they believe will receive no further short or long-term benefit of continuing. However we gave no guidance on a minimum number of sessions prior to discharge.

The proposed trial will provide participants allocated to the offer of acupuncture a course of up to twelve 50-minute sessions, usually weekly initially and fortnightly towards the end of the series. We expect the acupuncture to be delivered within a five-month period.

Participants allocated to the offer of Alexander Technique lessons will attend up to twenty 30-minute lessons. Generally, lessons will be weekly, with the option of being twice weekly initially and fortnightly towards the end of the series. We expect the lessons to be completed within five months.

Proposed frequency and duration of follow-up

Questionnaire-based data will be collected at baseline, and then at 3, 6 and 12 months post-randomisation. For the first six months we will also collect a single fortnightly pain score by text message (SMS), then monthly until 12 months, so that we can plot the trajectory of any change over this period.

Primary outcome measures

The primary outcome measured at baseline, 3, 6 and 12 months will be the Northwick Park Neck Pain Questionnaire [27], with the primary endpoint at 12 months. We used this outcome measure in both our physiotherapy for neck pain trial [24] and our acupuncture for neck pain pilot [6]. A secondary time point will be at 6 months, and an area-under-curve analysis will include 3, 6 and 12 month time-points.

Secondary outcome measures

Secondary outcome measures will be the Short Form (SF) -12v2 [28] for quality of life [28], and EQ-5D [29]

as health utility measure, both completed at baseline, 6 and 12 months. A measure of present pain intensity will be collected fortnightly by text message for the first six months of the trial, and monthly thereafter until the 12 months' endpoint. Participants who consent to respond with text messages will receive £5 as an up-front payment to cover costs. Adverse event data and beneficial effects which may be related to the intervention will be collected at 3, 6 and 12 months. Additionally, throughout the trial we will collect data on all adverse events using the Standard Operating Procedures of the York Trials Unit.

Treatment/intervention effect modifiers

A treatment/intervention effect modifier is a variable that provides an interaction, such that the effect of X on Y depends on the level of this additional variable. No causal sequence is implied by an interaction. For example, an intervention may be successful for males but not for females. This is called an interaction effect. To explore potential treatment/intervention effect modifiers, we will collect data at baseline on the participants' age, gender, education and employment, income band, and ethnicity as well as preferences, expectations and beliefs about the interventions. We will also collect preference data at final follow-up to examine if these change post-intervention.

Treatment/intervention effect mediators

A treatment/intervention effect mediator involves a variable that implies a causal sequence, intervention → mediator → outcome. We will add self-report measures that are designed to quantitatively explore mediators that might contribute to longer term change. At baseline, 6 and 12 months we will collect data on the following potential mediator variables:

1) self-efficacy, using the five-question pain management sub-scale of the Chronic Pain Self-Efficacy Scale [30], modified to be consistent with subsequent changes to the original scale by replacing the 0 to 10 scale with 0 to 8 and replacing 'certain' with 'confident' [31,32]. At 6 and 12 months we will add the question, 'Can you use/apply things you have learned from the intervention/care in everyday life situations to reduce pain?', a question modified from one used to assess self-management in a previous neck pain trial [33]. Consistent with data collected in previous research into unanticipated benefits of complementary therapies for back pain[34], we will also add at 6 and 12 months the questions, 'What effect, if any, has the treatment/care you have received for your neck pain had on you in the last 6 months? For example this might include your thoughts, feelings, reactions or activities' and 'Have you changed anything you do in the last 6 months as a result of the treatment/care you have received?'
2) stress management, using the four-question Perceived Stress Scale (PSS) [35,36] because psychosocial stressors are linked to musculoskeletal problems [37], and in particular mental stress increases the risk of having neck pain [38]. Related to this we will measure 'flow', using the two-question General Flow Index [39], as flow is considered a mechanism in traditional Chinese medicine for reducing stress-related conditions [40] and teachers report that flow is facilitated by lessons in the Alexander Technique.
3) incorporation of practitioner advice about self-care and/or life-style. We build on evidence on the role of self-care advice associated with acupuncture treatment [7] and Alexander lessons by asking, 'During the treatment/care you received in the last 6 to 12 months, did you learn to improve the way you live and care for yourself?', and 'If you answered yes, what were the changes that you were advised or taught how to make?'(open question), and 'To what extent are you able to put into practice the advice or teaching you received?'(scale 0 to 100), and 'To what extent are the changes you have been making helpful to you?'(scale 0 to 100).

Cost effectiveness

The economic evaluation will be undertaken primarily using an NHS perspective, as recommended by the National Institute for Health and Care Excellence (NICE) [41], and secondarily using a societal perspective. In all three arms we will collect data on health service resource use, including hospital and GP visits and medication at baseline, 6 and 12 months. We will use a resource-use questionnaire tailored to include services typically used by this population. We will seek the consent of participants to access their medical records and if needed check with individuals' GPs with regard to consultations and interventions. Quantities of services used will be multiplied by national unit cost estimates to estimate cost profiles for patients in the trial. We will also collect data on private health care used (including acupuncture, Alexander Technique lessons and other private care), as well as productivity related costs, such as days off work due to neck pain. The cost of productivity changes will be presented separately in accordance with NICE guidelines.

The base case evaluation will combine intervention-related costs and wider NHS costs with changes in QALYs, derived from EQ-5D, to perform a cost-utility analysis in the format recommended by NICE. We will estimate incremental cost-effectiveness at 12 months by

calculating the cost and QALY differences in the active intervention arms over and above the control. Cost and outcome data will be bootstrapped to adjust for skewness in the trial data. Data generated from the bootstrapping will be used to generate cost-effectiveness acceptability curves to demonstrate uncertainty around the cost-effectiveness ratio, based on different monetary values being attached to QALYs. In addition, as utility measures can be relatively insensitive to smaller changes in health status [42], we will use the NPQ as a secondary outcome measure.

Exploratory analyses drawing on patient preferences will be conducted to observe whether cost-effectiveness differs by the preference participants expressed for the interventions prior to randomisation.

Qualitative substudy

The focus of our qualitative study will add value to the trial by:

1) Adding depth to the interpretation of the main findings of the trial;
2) Gaining new understanding over and above the quantitative trial findings by focusing on the process of the intervention from the perspectives of key stakeholders;
3) Exploring practice and policy issues relevant to implementation of the trial results.

Qualitative material will be collected from participants at two time points and from practitioners at one time point. The purpose is to capture participants' experience of the intervention, which will be connected to their broader illness experience. We will address the impact of the interventions beyond reduction in pain, including influence on illness perception; health-related quality of life; and engagement in social activities and relationships.

Our methods involve the following:

1) We will conduct three focus group discussions with approximately eight participants (24 approximately in total) drawn from a general population with chronic neck pain. . These focus groups, which will involve people not involved in the trial, are designed to enable us to understand people's initial perceptions about the social consequences of neck pain, the relative merits and initial perceptions of the interventions, and the potential of these interventions for implementation as referral options in primary care. These discussions with nonparticipants are valuable in their own right and will also generate valuable general themes, which we will be able to explore in more detail during individual interviews. We will advertise for participants.
2) We will conduct in-depth interviews with ten trial participants from each of the three arms. There will be two interviews for each patient, one toward the end of the intervention (approximately 40 to 60 minutes) and a second more focussed one at 12 months (approximately 20 to 30 minutes). The two interviews will capture the participants' perceptions and experiences of their intervention, and the factors they associate with its impact over the short and longer term. The total interviews will number 60. Supplementary written qualitative material will also be sought from all remaining trial participants, by including an open question at the end of their 12-month questionnaire.
3) We will conduct three focus group discussions involving practitioners who are involved in the trial, one for acupuncturists, one for Alexander teachers, and one for general practitioners. These will be conducted during the intervention and follow-up phase of the trial, with approximately eight practitioners in each group. Our aim is to explore practitioner experiences and perspectives with a view to better understanding the implications if the interventions were implemented routinely in primary care.

Our approach to analysis will be iterative and aided by the use of Atlas.ti (version 5.2)software (Science Plus Group, Groningen, The Netherland, www.scienceplus.com). Initially, focus group discussions and interview material will be transcribed and organised according to analytical headings. During the analysis, regular meetings will be held between the research team to discuss the emergent themes from the fieldwork material. We will also be looking for potential points of comparison with the trial data. Focus group analysis will specifically concentrate on drawing out normative values and assumptions associated with trait identification. For the interviews, we will begin by identifying themes, which are first, interrogated in relation to each individual account, as a means of understanding a particular case; second, compared across cases by highlighting potential similarities and differences; and finally, related to those characteristics of the respondent that could be reasonably justified as an explanation which mediated experience. Our analysis will also benefit from any insights gained from the trial data and in particular look for ways of explaining some of the more quantitative findings. The meaning people give to the intervention, for example, will provide valuable material on how any intervention will work when faced with the 'messiness' of practice.

Proposed sample size and a description of the power calculations

We will recruit 500 participants in total to the trial. Our sample size calculations were performed for a simple

comparison of two groups. From our pilot, the standard deviation of the primary endpoint, the Northwick Park Questionnaire (NPQ), was 16%, and the correlation between baseline and 3-month NPQ was 0.69 [6]. A clinically meaningful difference on the NPQ is 5 percentage points [43], a 0.31 effect size. With 90% power and alpha = 5% we would require 113 participants per arm after adjusting for baseline. We are aiming for a loss to follow-up of 20% but in our estimate of sample size we are conservative, allowing for 30% loss to follow-up. Randomising into three equally sized groups, our required total sample size will be 500.

For our primary comparison, we will estimate the difference in mean score from the NPQ at 12 months between the acupuncture and control groups and between the Alexander Technique lessons and control groups, after adjustment for baseline pain score using analysis of covariance. Each of our two active interventions tested against control can be interpreted quite independently of the other. This does not constitute a problem of multiple testing, as there might be if we had two different kinds of acupuncture. If we find acupuncture to be significantly better than the control, then we would conclude that it worked. But this test would tell us nothing about Alexander Technique lessons versus control. Therefore when testing two independent hypotheses, we do not need to use multiple comparison procedures. We will, as a secondary analysis, estimate the clinical difference between acupuncture and Alexander Technique lessons.

Planned recruitment methods

We will use our well-established 'database' recruitment method. We will recruit GP practices local to areas where there is sufficient capacity from acupuncturists and Alexander teachers. Potentially suitable participants will be identified from electronic medical records held by participating GP practices. This will involve searching for primary care consultations coded as related to neck pain, using pre-specified READ codes, and will be performed by practice staff. Only those patients who have consulted for neck pain three months or more before the database search will be invited to take part in the trial. Patients who have not consulted for neck pain, or have only done so for the first time within the preceding three-month period, will be excluded from the study. The lead GP within each practice will then be given the opportunity to exclude any unsuitable patients on the resulting list prior to information being sent to patients about the trial. General practices will then mail out patient information leaflets, baseline questionnaires and consent forms. The letter will ask patients if they still have problems with neck pain and if so, will invite them to complete the consent form and baseline questionnaire that includes the Northwick Park Questionnaire and to return these to the York Trials Unit. Questionnaires will be screened by the research team and a list compiled of all those who appear eligible to take part. This list will then be sent back to the lead GP for final approval, at which point the patient's medical records will be examined in detail. Those who are subsequently approved will then be randomised using a secure method, and a letter sent to their GPs stating to which arm of the trial they have been allocated. Participants allocated to one of the active interventions will be sent a letter and invited to phone the office to arrange an initial appointment with a practitioner at a time and location that is suitable. A confirmation letter is then sent to the patient providing details of the appointment, and a logbook is sent to the practitioner.

In addition we will identify potential participants who respond to advertisements in GP practices. Patients interested in taking part in the study will be asked to phone the trial team on a free phone number for further information. If the patient is still interested in taking part, we will record the patient's name, contact details and name of their GP practice on a secure database with restricted access, and send out a pack inviting them to join the study. This pack will include the participant information sheet, a baseline questionnaire, two consent forms (that is, the same documents sent to patients identified from the database search), a covering letter, and prepaid envelopes for return of completed consent form and questionnaire. Upon receipt, the questionnaire and consent form will be dealt with in the same way as those received from patients identified in a database search (see above).

In our neck pain pilot based in York we used this approach with one general practice with a list size of 15,694 patients, from which 227 had consulted with neck pain in the previous year [6]. Of these, 28 (12.3%) consenting patients were eligible to participate in the pilot and 24 (10.5%) were recruited. Since eligibility criteria are more restrictive in this trial (that is, minimum chronicity of three months) the proportion of volunteers deemed to be suitable at the point of secondary screening is likely to be less than that observed in the pilot study. However we are removing the cut-off of one year since previous consultation with neck pain, which in turn will support an increase in the number of volunteers per practice. We estimate a GP practice with a list size of 10,000 patients will identify 300 patients eligible for an invitation to participate in the trial. Of these, 5% are predicted to sign consent forms and be judged suitable when their baseline questionnaire is screened. We therefore expect to recruit on average 15 participants per practice from 33 general practices over a 12-month period.

Attendance rates and loss to follow-up

Less than optimal attendance at sessions, as discussed above, is an inevitable part of a pragmatic trial given that

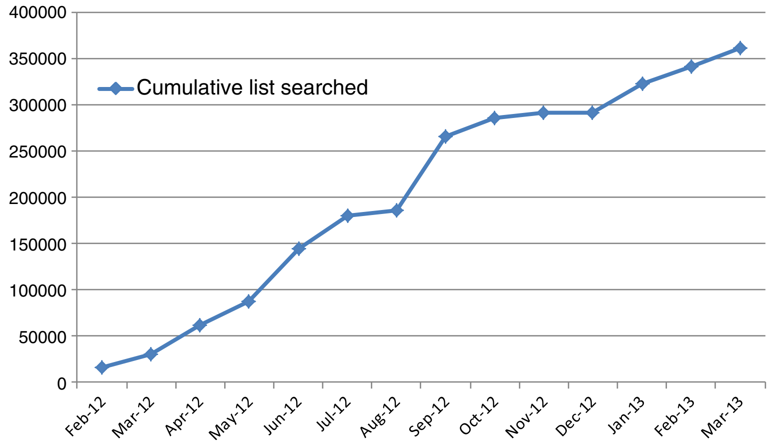

Figure 1 General practice database searches by patient population.

our aim is to evaluate everyday practice. Early termination of an intervention can be a sign that it has worked, and that the neck pain is alleviated. Alternatively early discontinuation due to perceived lack of results may occur. To optimise attendance, practitioners will be selected for their training and experience, and will also receive induction into trial processes. Our experience with acupuncture trials is that 8 out of 10 sessions were taken up with our back pain trial [9] and 9 out of 10 sessions for irritable bowel syndrome [44]. Similar data were found from the Alexander Technique lessons for the back pain trial [13].

Loss to follow-up (of self-report questionnaire data) will be minimised by a number of means, including having the research team highly organised and efficient in handling all matters that affect participants. Questionnaires will be sent out in a timely manner along with text message reminders. Where a questionnaire is not returned, reminders will be sent out. If the reminders fail to get a response, a telephone call will be made (by a researcher blinded to group allocation) to collect the data for the primary outcome measure and the EQ-5D. In our experience of postal questionnaires with additional follow-up as described above in a trial of acupuncture for low back pain, we found response rates of over 93% in the acupuncture group and 83% in the usual care group at 12 months [9]. Similar response rates were found in the Alexander Technique lessons for back pain trial [13]. All participants will receive £5 with their final questionnaire, which is likely to encourage a higher return rate.

A summary of the statistical analysis plan

Analysis will be by intention-to-treat. All of those randomly allocated will remain in their group for analytical purposes

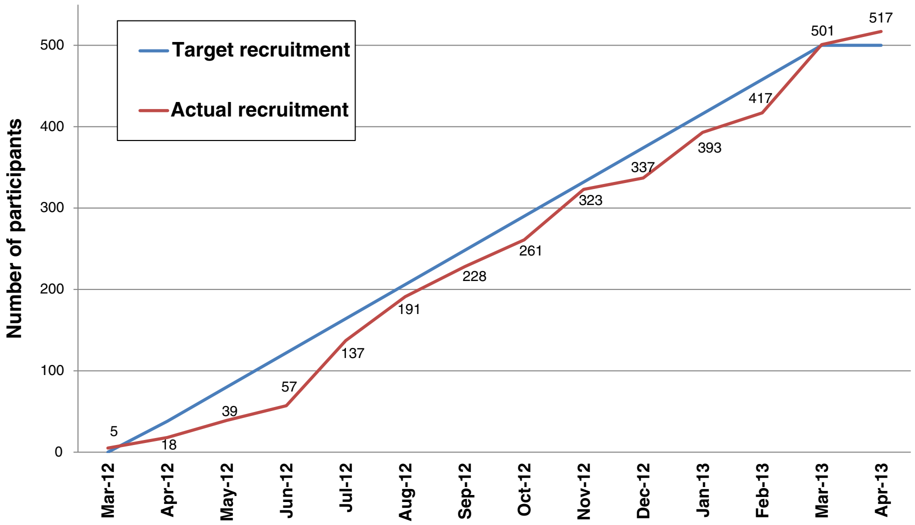

Figure 2 Patient recruitment – final data (updated subsequent to submission of manuscript).

irrespective of whether or not they actually take up or fully adhere with the intervention. Statistically we will use analysis of covariance to control for baseline values. Multiple imputation will be used to account for missing values.

As this is a pragmatic trial, participants are offered, but may not receive a full course of sessions or lessons. Some may discontinue if they get better before the end of their course. Others may discontinue if they perceive no benefit. In a trial of Alexander Technique lessons for low back pain patients, average attendance was either 5 out of 6 offered lessons, or 20 out of 24 lessons offered [13]. We will incorporate Complier Average Causal Effect (CACE) analysis to control for any dilution effects of people not taking up their allocated intervention [45]. We will also ascertain whether there is a relationship between levels of attendance and outcome, and whether outcomes are better or not for those participants who have attended at least 75% of sessions.

To evaluate trajectories of change from the text message scores, we will fit a common curve for each group using polynomial regression. We will use a polynomial within-subject, by analysis of covariance, to enable us to plot an empirical curve for each group. We can compare the three groups statistically to test for interactions between intervention group and time. Moreover these text scores will provide useful data for multiple imputation for missing data.

We will use regression models to explore whether preference, belief or expectation are treatment effect modifiers. We will explore whether potential treatment effect mediators (self-efficacy, stress management, incorporation of advice about self-care and lifestyle) might be associated with longer term change.

We will investigate variations in outcomes between practitioners, which we call practitioner effects, in a separate analysis using the 'artificial cluster' method. Each control participant will hypothetically be allocated a practitioner, so that each practitioner is linked to a cluster of both intervention and control participants. For each practitioner the difference in mean NPQ score between those allocated to the intervention and those allocated control will be found and a confidence interval for the difference found by the t-method. Adjustment for baseline NPQ will be done using analysis of covariance on mean baseline NPQ for the cluster and using baseline minus outcome differences.

Outcome data will be examined to determine if it influences subsequent preferences by comparing participants' pre-randomisation preferences to those given at 12 months post-randomisation.

Discussion

The proposed research received ethics approval from Leeds West Research Ethics Committee (11/YH/0402). All intervention sites involved in the trial obtained 'site specific approval' from the NHS research ethics committee. The first patient was randomised on 22 March 2102 in York, followed by Leeds, Sheffield and Manchester. In total, 33 GP practices participated in the recruitment process, with over 350,000 of their patient records screened, see Figure 1. Patient recruitment at the time of submission (5 April 2013) has reached 501 participants, see Figure 2, and we expect to recruit a further 20 participants before recruitment is closed at the end of April 2013.

Trial status

Recruitment is open at the time of submission.

Abbreviations

ATLAS: Alexander technique lessons, acupuncture sessions; CASE: Complier average causal effect; GP: General practitioner; NHS: National health service; NICE: National institute for health and care excellence; NPQ: Northwick park neck pain questionnaire; PSS: Perceived stress scale; QALY: Quality adjusted life year; SMS: Short message service; STAT,: Society of teachers of the alexander technique; TCM: Traditional chinese medicine.

Competing interests

The authors declare that they have no competing interests.

Authors' contributions

HM as Chief Investigator, KA, KB, MB, SP, DT and IW were co-applicants on the grant application to Arthritis Research UK, and were involved in the design of the project and its implementation, as was JW. HT and SR are the trial managers, supported by JE and AH. MB and UM are the trial statisticians. SP and HE are the trial's health economists. JW/KB and HL have liaised with and supported the Alexander Technique teachers and acupuncturists respectively. IW has provided a clinician perspective on adverse events and their reporting. DT as Director of the York Trials Unit has supported and advised the trial management. All authors have agreed to the final version of the protocol. All authors read and approved the final manuscript.

Acknowledgements

We wish to acknowledge contributions from all the participants and practitioners as well as the following: Matthew Bailey, Sarah Brown, Sue Collins, Cindy Cooper, Ben Cross, David Geddes, Pauline Holloway, Gareth Jones, Elaine Hay, David Laverack, Tony Murray, Lucy Revell, Deborah Sharp and Val Wadsworth.

Funding body

The ATLAS trial has been funded by Arthritis Research UK, grant reference: Grant Ref 19702. The funding body had some involvement, via Arthritis Research UK's Musculoskeletal Pain Clinic Studies Group, in developing the original design of the study prior to providing the grant. The funding body has no input into the collection, analysis, and interpretation of data; in the writing of the manuscript; nor in the decision to submit the manuscript for publication.

Author details

[1]Department of Health Sciences, University of York, York YO10 5DD, UK. [2]Society of Teachers of the Alexander Technique, 1st Floor, Linton House, 39-51, Highgate Road, London NW5 1RS, UK. [3]Department of Health Sciences and Hull York Medical School, University of York, York YO10 5DD, UK.

Received: 8 April 2013 Accepted: 25 June 2013
Published: 10 July 2013

References

1. Binder AI: **Cervical spondylosis and neck pain.** *BMJ* 2007, **334**:527–531.
2. Ferrari R, Russell AS: **Neck pain.** *Best Pract Res Clin Rheumatol* 2003, **17**:57–70.
3. Linton SJ, Ryberg M: **Do epidemiological results replicate? The prevalence and health-economic consequences of neck and back pain in the general population.** *Eur J Pain* 2000, **4**:347–354.
4. Hurwitz EL, Goldstein MS, Morgenstern H, Chiang LM: **The impact of psychosocial factors on neck pain and disability outcomes among primary care patients: results from the UCLA Neck Pain Study.** *Disabil Rehabil* 2006, **28**:1319–1329.

5. National Collaborating Centre for Mental Health: *The NICE guideline on the treatment and management of depression in adults 2010 p. 1–707.* Available from: http://www.nice.org.uk/nicemedia/live/12329/45896/45896.pdf.
6. Salter GC, Roman M, Bland MJ, MacPherson H: **Acupuncture for chronic neck pain: a pilot for a randomised controlled trial.** *BMC Musculoskelet Disord* 2006, **7**:99.
7. MacPherson H, Thomas K: **Self-help advice as a process integral to traditional acupuncture care: implications for trial design.** *Complement Ther Med* 2008, **16**:101–106.
8. Yardley L, Dennison L, Coker R, Webley F, Middleton K, Barnett J, Beattie A, Evans M, Smith P, Little P: **Patients' views of receiving lessons in the Alexander Technique and an exercise prescription for managing back pain in the ATEAM trial.** *Fam Pract* 2010, **27**:198–204.
9. Thomas KJ, MacPherson H, Thorpe L, Brazier J, Fitter M, Campbell MJ, Roman M, Walters SJ, Nicholl J: **Randomised controlled trial of a short course of traditional acupuncture compared with usual care for persistent non-specific low back pain.** *BMJ* 2006, **333**:623–626.
10. Vickers AJ, Rees RW, Zollman CE, McCarney R, Smith CM, Ellis N, Fisher P, Van Haselen R: **Acupuncture for chronic headache in primary care: large, pragmatic, randomised trial.** *BMJ* 2004, **328**:744.
11. Ratcliffe J, Thomas KJ, MacPherson H, Brazier J: **A randomised controlled trial of acupuncture care for persistent low back pain: cost effectiveness analysis.** *BMJ* 2006, **333**:626–628.
12. Wonderling D, Vickers AJ, Grieve R, McCarney R: **Cost effectiveness analysis of a randomised trial of acupuncture for chronic headache in primary care.** *BMJ* 2004, **328**:747.
13. Little P, Lewith G, Webley F, Evans M, Beattie A, Middleton K, Barnett J, Ballard K, Oxford F, Smith P, Yardley L, Hollinghurst S, Sharp D: **Randomised controlled trial of Alexander technique lessons, exercise, and massage (ATEAM) for chronic and recurrent back pain [see comment].** *BMJ* 2008, **337**:a884.
14. Hollinghurst S, Sharp D, Ballard K, Barnett J, Beattie A, Evans M, Lewith G, Middleton K, Oxford F, Webley F, Little P: **Randomised controlled trial of Alexander technique lessons, exercise, and massage (ATEAM) for chronic and recurrent back pain: economic evaluation.** *BMJ* 2008, **337**:a2656.
15. National Institute for Health and Clincal Excellence: *Low back pain. Early management of persistent non-specific low back pain.* London, UK: National Institute for Health and Clinical Excellence; 2009.
16. Witt CM, Jena S, Brinkhaus B, Liecker B, Wegscheider K, Willich SN: **Acupuncture for patients with chronic neck pain.** *Pain* 2006, **125**:98–106.
17. Willich SN, Reinhold T, Selim D, Jena S, Brinkhaus B, Witt CM: **Cost-effectiveness of acupuncture treatment in patients with chronic neck pain.** *Pain* 2006, **125**:107–113.
18. Paterson C, Dieppe P: **Characteristic and incidental (placebo) effects in complex interventions such as acupuncture.** *Br Med J* 2005, **330**:1202–1205.
19. Jones FP: *Freedom to change: Development and science of the Alexander Technique.* London: Mouritz; 1997.
20. MacPherson H, Richmond S, Bland JM, Lansdown H, Hopton A, Kang'ombe A, Morley S, Perren S, Spackman E, Spilsbury K, Torgerson D, Watt I: **Acupuncture, Counseling, and Usual care for Depression (ACUDep): study protocol for a randomized controlled trial.** *Trials* 2012, **13**:209.
21. Klaber Moffett JA, Jackson DA, Richmond S, Hahn S, Coulton S, Farrin A, Manca A, Torgerson DJ: **Randomised trial of a brief physiotherapy intervention compared with usual physiotherapy for neck pain patients: outcomes and patients' preference.** *BMJ* 2005, **330**:75.
22. MacPherson H, Thomas K, Walters S, Fitter M: **The York acupuncture safety study: prospective survey of 34 000 treatments by traditional acupuncturists.** *Br Med J* 2001, **323**:486–487.
23. MacPherson H, Scullion A, Thomas KJ, Walters S: **Patient reports of adverse events associated with acupuncture treatment: a prospective national survey.** *Qual Saf Health Care* 2004, **13**:349–355.
24. Trinh K, Graham N, Gross A, Goldsmith CH, Wang E, Cameron ID, Kay T, Cervical Overview Group: **Acupuncture for neck disorders. 2006. Cervical Overview Group.** *Cochrane Database Syst Rev* 2006, **3**:CD004870.
25. Gross A, Miller J, D'Sylva J, Burnie SJ, Goldsmith CH, Graham N, Haines T, Brønfort G, Hoving JL: **Manipulation or mobilisation for neck pain: a Cochrane Review.** *Man Ther* 2010, **15**:315–333.
26. Moffett JK, Jackson DA, Gardiner ED, Torgerson DJ, Coulton S, Eaton S, Mooney MP, Pickering C, Green AJ, Walker LG, May S, Young S: **Randomized trial of two physiotherapy interventions for primary care neck and back pain patients: 'McKenzie' vs brief physiotherapy pain management.** *Rheumatology (Oxford)* 2006, **45**:1514–1521.
27. Leak AM, Cooper J, Dyer S, Williams KA, Turner-Stokes L, Frank AO: **The Northwick Park Neck Pain Questionnaire, devised to measure neck pain and disability.** *Br J Rheumatol* 1994, **33**(5):469–474.
28. Ware J Jr, Kosinski M, Keller SD: **A 12-Item short-form health survey: construction of scales and preliminary tests of reliability and validity.** *Med Care* 1996, **34**:220–233.
29. Kind P: **The EuroQoL instrument: an index of health-related quality of life.** In *Quality of Life and Pharmacoeconomics in Clinical Trials.* Edited by Spilker B. Philadelphia: Lippincott-Raven; 1996:191–201.
30. Anderson KO, Dowds BN, Pelletz RE, Thomas EW, Peeters-Asdourian C: **Development and initial validation of a scale to measure self-efficacy beliefs in patients with chronic pain.** *Pain* 1995, **63**:77–83.
31. Woby SR, Roach NK, Urmston M, Watson PJ: **The relation between cognitive factors and levels of pain and disability in chronic low back pain patients presenting for physiotherapy.** *Eur J Pain* 2007, **11**:869–877.
32. Thompson DP, Urmston M, Oldham JA, Woby SR: **The association between cognitive factors, pain and disability in patients with idiopathic chronic neck pain.** *Disabil Rehabil* 2010, **32**:1758–1767.
33. Gustavsson C, Denison E, von Koch L: **Self-management of persistent neck pain: A randomized controlled trial of a multi-component group intervention in primary health care.** *Eur J Pain* 2010, **14**:630.
34. Hsu C, Bluespruce J, Sherman K, Cherkin D: **Unanticipated benefits of CAM therapies for back pain: an exploration of patient experiences.** *J Altern Complement Med* 2010, **16**:157–163.
35. Cohen S, Kamarck T, Mermelstein R: **A global measure of perceived stress.** *J Health Soc Behav* 1983, **24**:385–396.
36. Mitchell AM, Crane PA, Kim Y: **Perceived stress in survivors of suicide: psychometric properties of the perceived stress scale.** *Res Nurs Health* 2008, **31**:576–585.
37. Houtman IL, Bongers PM, Smulders PG, Kompier MA: **Psychosocial stressors at work and musculoskeletal problems.** *Scand J Work Environ Health* 1994, **20**:139–145.
38. Korhonen T, Ketola R, Toivonen R, Luukkonen R, Hakkanen M, Viikari-Juntura E: **Work related and individual predictors for incident neck pain among office employees working with video display units.** *Occup Environ Med* 2003, **60**:475–482.
39. Bakker AB: **The work-related flow inventory: Construction and initial validation of the WOLF.** *J Vocat Behav* 2008, **72**:400–414.
40. Maciocia G: *The Foundations of Chinese Medicine.* Edinburgh: Churchill Livingstone; 1989.
41. National Institute for Health and Clinical Excellence: *Guide to the methods of technology appraisal.* London: National Institute for Health and Clinical Excellence; 2008.
42. Torgerson DJ, Rafferty J: **Economic notes: measuring outcomes in economic evaluations.** *BMJ* 1999, **318**:1413.
43. Dziedzic K, Hill J, Lewis M, Sim J, Daniels J, Hay EM: **Effectiveness of manual therapy or pulsed shortwave diathermy in addition to advice and exercise for neck disorders: a pragmatic randomized controlled trial in physical therapy clinics.** *Arthritis Rheum* 2005, **53**:214–222.
44. MacPherson H, Bland M, Bloor K, Cox H, Geddes D, Kang'ombe A, Reynolds J, Stamuli E, Stuardi T, Tilbrook H, Torgerson D, Whorwell P: **Acupuncture for irritable bowel syndrome: a protocol for a pragmatic randomised controlled trial.** *BMC Gastroenterol* 2010, **10**:63.
45. Hewitt CE, Torgerson DJ, Miles JN: **Is there another way to take account of noncompliance in randomized controlled trials?** *CMAJ* 2006, **175**:347.

doi:10.1186/1745-6215-14-209
Cite this article as: MacPherson *et al.*: **Alexander Technique Lessons, Acupuncture Sessions or usual care for patients with chronic neck pain (ATLAS): study protocol for a randomised controlled trial.** *Trials* 2013 **14**:209.
